# A putative RNA-interference-based immune system in prokaryotes: computational analysis of the predicted enzymatic machinery, functional analogies with eukaryotic RNAi, and hypothetical mechanisms of action

**DOI:** 10.1186/1745-6150-1-7

**Published:** 2006-03-16

**Authors:** Kira S Makarova, Nick V Grishin, Svetlana A Shabalina, Yuri I Wolf, Eugene V Koonin

**Affiliations:** 1 National Center for Biotechnology Information, National Library of Medicine, National Institutes of Health, Bethesda, MD 20894, USA; 2 Department of Biochemistry, University of Texas Southwestern Medical Center, 5323 Harry Hines Blvd, Dallas, TX 75390-9050, USA

## Abstract

**Background:**

All archaeal and many bacterial genomes contain Clustered Regularly Interspaced Short Palindrome Repeats (CRISPR) and variable arrays of the CRISPR-associated (*cas*) genes that have been previously implicated in a novel form of DNA repair on the basis of comparative analysis of their protein product sequences. However, the proximity of CRISPR and *cas *genes strongly suggests that they have related functions which is hard to reconcile with the repair hypothesis.

**Results:**

The protein sequences of the numerous *cas *gene products were classified into ~25 distinct protein families; several new functional and structural predictions are described. Comparative-genomic analysis of CRISPR and *cas *genes leads to the hypothesis that the CRISPR-Cas system (CASS) is a mechanism of defense against invading phages and plasmids that functions analogously to the eukaryotic RNA interference (RNAi) systems. Specific functional analogies are drawn between several components of CASS and proteins involved in eukaryotic RNAi, including the double-stranded RNA-specific helicase-nuclease (dicer), the endonuclease cleaving target mRNAs (slicer), and the RNA-dependent RNA polymerase. However, none of the CASS components is orthologous to its apparent eukaryotic functional counterpart. It is proposed that unique inserts of CRISPR, some of which are homologous to fragments of bacteriophage and plasmid genes, function as prokaryotic siRNAs (psiRNA), by base-pairing with the target mRNAs and promoting their degradation or translation shutdown. Specific hypothetical schemes are developed for the functioning of the predicted prokaryotic siRNA system and for the formation of new CRISPR units with unique inserts encoding psiRNA conferring immunity to the respective newly encountered phages or plasmids. The unique inserts in CRISPR show virtually no similarity even between closely related bacterial strains which suggests their rapid turnover, on evolutionary scale. Corollaries of this finding are that, even among closely related prokaryotes, the most commonly encountered phages and plasmids are different and/or that the dominant phages and plasmids turn over rapidly.

**Conclusion:**

We proposed previously that Cas proteins comprise a novel DNA repair system. The association of the *cas *genes with CRISPR and, especially, the presence, in CRISPR units, of unique inserts homologous to phage and plasmid genes make us abandon this hypothesis. It appears most likely that CASS is a prokaryotic system of defense against phages and plasmids that functions via the RNAi mechanism. The functioning of this system seems to involve integration of fragments of foreign genes into archaeal and bacterial chromosomes yielding heritable immunity to the respective agents. However, it appears that this inheritance is extremely unstable on the evolutionary scale such that the repertoires of unique psiRNAs are completely replaced even in closely related prokaryotes, presumably, in response to rapidly changing repertoires of dominant phages and plasmids.

**This article was reviewed by**: Eric Bapteste, Patrick Forterre, and Martijn Huynen.

**Open peer review:**

Reviewed by Eric Bapteste, Patrick Forterre, and Martijn Huynen.

For the full reviews, please go to the Reviewers' comments section.

## Background

The discovery of the elaborate and versatile systems of RNA silencing in eukaryotes is one of the pivotal advances in biology of the last decade [1-6]. There are two major, distinct forms of regulatory small RNAs involved in eukaryotic gene silencing: small interfering (si) RNAs and micro (mi) RNAs. siRNAs are produced from double-stranded RNAs of viruses and transposable elements, which are processed by the dicer nuclease, one of the essential components of the RNA-Induced Silencing Complexes (RISCs) [7-9]. Dicer cleaves long dsRNA molecules into short, 21–22 nucleotide duplexes which are subsequently unwound by the RISC to yield mature siRNAs. The RISC-siRNA complex then binds to the target mRNA which is cleaved by the slicer nuclease, another crucial component of RISC, to release the RISC-siRNA which acts as a recyclable catalyst [9,10]. In addition to silencing genes of exogenous agents, a distinct class of longer, 28 nt siRNAs, the so-called repeated-associated siRNAs (rasiRNAs), silence expression of chromosomal copies of transposons and transposon-like repeats [11-13].

Unlike the siRNAs, 21–25 nt-long miRNAs are encoded in eukaryotic genomes and are either perfectly (in plants) or imperfectly (in animals) complementary to sequences in the 3'-untranslated regions of specific endogenous mRNAs [6,13]. Base-pairing of miRNAs with the target mRNAs, which is mediated by a distinct form of RISC, results either in RNA cleavage or in down-regulation of translation [8]. Evidence is rapidly accumulating that numerous, probably, thousands of miRNAs in animals and plants are major players in development regulation and chromatin remodeling [6].

Prokaryotes have apparent functional counterparts to the miRNA system, i.e., regulation of bacterial gene expression by small antisense RNAs. The best characterized of these pathways employ the RNA-binding protein Hfq for small RNA presentation and RNAse E for target degradation [14-17]. *Escherichia coli *has ~60 microRNA genes, and comparable numbers of expressed, small antisense RNAs have been detected in the archaea *Archaeoglobus fulgidus *[18] and *Sulfolobus solfataricus *[19], suggesting an important role of this regulatory mechanism in prokaryotic physiology. In addition, small antisense RNAs have been shown to regulate plasmid replication and killing of plasmid-free bacterial cells by silencing specific plasmid genes [20-22]. In contrast, counterparts to the eukaryotic siRNA mechanism so far have not been described in prokaryotes. Here, we apply comparative genomics and in-depth computational analysis of protein and RNA sequences and structures to predict a distinct prokaryotic siRNA-like system and the associated enzymatic apparatus.

In a previous comparative-genomic study, which has been originally conceived as a test case for methods for conserved gene neighborhood analysis we have developed, we characterized an extensive set of genes that included several proteins related to DNA or RNA metabolism and was, mostly, specific to thermophiles [23]. These genes comprise a complex array of overlapping neighborhoods that are partially conserved but highly diversified, in terms of both gene composition and gene order, and are represented in all archaeal and many bacterial genomes [23,24]. At the time of its discovery, we hypothesized that these genes encoded an uncharacterized, versatile repair system, largely, associated with the thermophilic lifestyle [23].

Independently and almost simultaneously, Jansen and coworkers found [25] that at least several genes from this gene neighborhood were tightly associated with the so-called Clustered Regularly Interspaced Short Palindrome Repeats (CRISPR); the acronym *cas *(for CRISPR-associated) genes was thus coined. The CRISPR are a distinct class of repetitive elements that are present in numerous prokaryotic genomes. A CRISPR element consists of a direct repeat of ~28–40 base pairs (bp), with the copies separated by a unique sequence of ~25–40 bp. Typically, CRISPR form tandem arrays containing from 4 to >100 elements. Most of the genomes contain a single array of CRISPR in which the sequences of the repeats are (nearly) identical; some, however, possess multiple CRISPR cassettes that may have substantially different sequences [24,25]. The repeats in CRISPR from different genomes show only limited similarity, but often retain distinct, conserved motifs shared even by distant species including archaea and bacteria [18,26]. There seems to be a strict link between CRISPR and cas genes, suggestive of a (nearly) mutualistic relationship: the great majority of the genomes that contain CRISPR also have at least a minimal set of cas genes, and vice versa.

Recently, Mojica and coworkers reported that the unique inserts in some of the CRISPR are homologous to fragments of bacteriophage and plasmid genes [27]. This led to the hypothesis that the CRISPR might have a function in the defense of prokaryotes against invading foreign replicons and that there could be functional analogies between this putative defense system and eukaryotic RNA interference. Similar findings have been independently reported by two other groups [28,29].

The recent rapid growth of the number and diversity of sequenced prokaryotic genomes has led to a dramatic increase in the complexity of the identified cas gene arrays [24]. Here we describe the results of an exhaustive sequence analysis of the Cas protein sequences which yielded a classification of these proteins, several new functional predictions, and a reconstruction of evolutionary relationship between these genes. We propose that the cas genes encode the protein machinery of a prokaryotic siRNA-like system that performs, primarily, but perhaps, not exclusively, defense functions and is generally similar, in some respects, to eukaryotic siRNA, and in other respects, to the vertebrate immune system. The predicted enzymatic machinery of this system seems to be functionally analogous, but not homologous, to the protein apparatus involved in the eukaryotic RNA-mediated gene silencing. Finally, we outline possible molecular mechanisms of the predicted prokaryotic siRNA system. The hypothesis on the involvement of Cas proteins in an RNAi-type mechanism supplants the previous proposal that these proteins might comprise a novel DNA repair system [23] which is hardly compatible with the tight association of these proteins with CRISPR and the existence of unique CRISPR inserts homologous to phage and plasmid sequences.

## Results and discussion

### Identification, classification and evolutionary analysis of cas genes

In the original study of the *cas *gene neighborhoods, which was performed with ~40 genomes, we identified ~20 protein families that were tightly or more loosely associated with the system we now call CASS. A recent update by Haft and coworkers with >200 genomes yielded a diverse "guild" of ~45 Cas protein families [24]. Many of the Cas proteins show very low sequence conservation which makes identification of homologous relationship between them a non-trivial task. We employed an iterative approach to the exhaustive analysis of the Cas protein sequences. The protein sequences of each Cas family were compared to the protein sequences from all available prokaryotic genomes using PSI-BLAST [30,31], the proteins encoded in the neighborhoods of all identified candidates were used as queries for further searches, and the process was iterated until convergence. The sequences of Cas proteins were, in addition, carefully compared to each other, in an attempt to identify possible traces of common origin of some of these genes that have so far eluded detection [for a complete list of Gene Identification (GI) numbers of the detected Cas proteins, see Additional file 1].

#### COG1518, a universal marker of CASS

In agreement with the previous observations [23], we found that Cas1 (COG1518 in the Clusters of Orthologous Group of proteins classification system [32]) is the best marker of the CRISPR-associated systems (CASS). This gene encodes a highly conserved protein and that is represented in all cas neighborhoods, with the single exception of *Pyrococcus abyssii*. A PSI-BLAST search for COG1518 members in the completely sequenced prokaryotic genomes revealed the presence of at least one representative of this COG in 77 of the 177 analyzed genomes.

**Figure 1:**
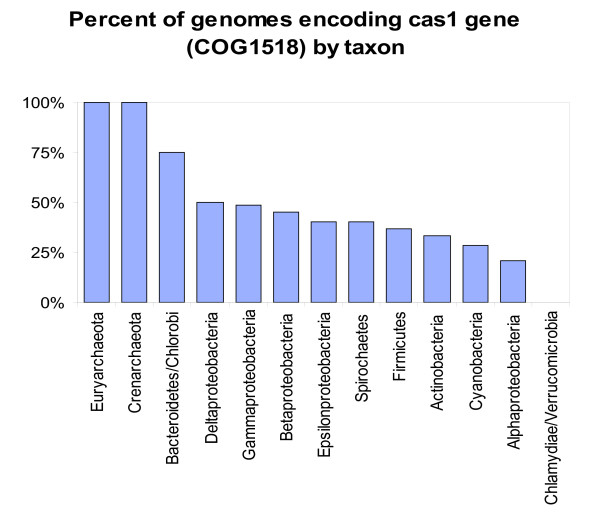
Distribution of COG1518 genes and, by implication, CASS among prokaryotic lineages.

Given that COG1518 is represented in all versions of CASS, the distribution of this gene among prokaryotic lineages serves as a proxy for the distribution of CASS itself (*P. abyssii *was included as having CASS, the absence of COG1518 notwithstanding); this distribution is, obviously, non-uniform (Fig. 1). All sequenced archaeal genomes, including, notably, the tiny genome of *Nanoarchaeum equitans*, encode this protein. In contrast, in each of the bacterial lineages with a representative set of sequenced genomes, half or more species do not have COG1518. We came across several cases of differences in the presence of CASS among closely related bacterial species and even between strains of the same species. Thus, *Corynebacterium diphtheriae *has a COG1518 gene but *Corynebacterium efficiens *does not; similarly, CASS was detected in *Mycobacterium tuberculosis *but not in *Mycobacterium bovis *or *Mycobacterium leprae*; significant differences in the composition of CASS were noticed even between strains of several bacterial species, e.g., *Thermus thermophilus *[33].

**Figure 2:**
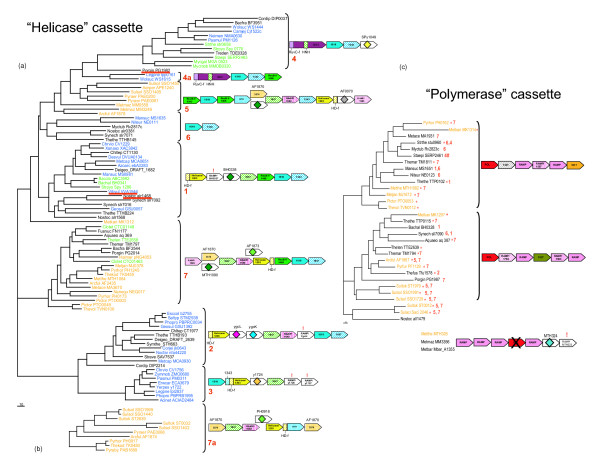
**Phylogenies of the key cas genes and organization of cas operons**. (a) Phylogenetic tree for COG1518 proteins (b) Phylogenetic tree for COG1203 proteins (predicted helicase) from the CASS versions lacking COG1518 (c) Phylogenetic tree for the predicted CASS polymerase (COG1353). Prokaryotic lineages are color-coded: orange, archaea; blue, Proteobacteria; green, low-GC Gram-positive bacteria; black, other bacteria. In the operon organizations cartoons, orthologous genes are color-coded and denoted by either the predicted function or the COG number. Exclamation points denote previously undetected RAMPs. The names of species that have a reverse transcriptase gene within one of the cas operons are underlined in red. In the left panel, the distinct versions of CASS are numbered, and in the right panel, these numbers are given at tree leaves to indicate the helicase cassette(s) that co-occurs with the given polymerase cassette.

Phylogenetic analysis of COG1518 combined with gene neighborhood comparisons yields a clear classification of CASS based on gene composition and (predicted) operon organization (Fig. 2a) which is, generally, in agreement with the recent study of Haft et al [24]. Altogether, we delineated 7 distinct versions of CASS which show substantial differences in gene composition and predicted operon organization. Only four CASS variants (CASS1-4) have a more or less stable operon organization. While a distinct operon organization motif is recognizable in the other CASS variants as well (Fig. 2a), these versions have undergone numerous, complex rearrangements in different prokaryotic genomes including gene duplications, fusion with other CRISPR-related operons, insertion of additional genes, and others. Thus, in line with the observation that CASS is often eliminated from genomes during evolution, this plasticity of CASS operon organization suggests that the CRISPR-associated genomic regions are "hot spots" of recombination and ensuing genome rearrangement. Taken together, all these observations demonstrate the extraordinary evolutionary mobility of CASS.

The recent work of Haft et al has identified a "guild" of over 45 protein families comprising the CASS [24]. Here we refine this classification by unifying many of the families into superfamilies and also expand the list of CASS-linked genes to include those that are found in *cas *operons less commonly and comprise the "cloud" surrounding the core CASS components. Only COG1518 and COG1343 (see below) are invariably present in all versions of CASS (with the exception of *P. abyssii *and several bacterial species that carry disrupted COG1518 gene). A few genomes that have a cassette of CRISPR-associated genes without COG1518 also possess another CRISPR-associated operon in a different genomic location that does include this gene. Altogether, we end up with ~25 gene families that are tightly associated with CASS (Table 1)

**Table 1:** Protein components of CASS

	**Family**	**Subfamily**^A^	**Phyletic distribution**^B^	**Comments**
1	COG1518	COG1518 (cas1)	All	Putative novel nuclease/integrase; Mostly α-helical protein
2	COG1343	COG1343 (cas2), COG3512, ygbF-like; MTH324-like; y1723_N-like;	All	Small protein related to VapD, fused to helicase (COG1203) in y1723-like proteins
3	COG1203	COG1203 (cas3)	All	DNA helicase; Most proteins have fusion to HD nuclease
4	RecB-like nuclease	COG1468 (cas4), COG4343	All	RecB-like nuclease; Contains three-cysteine C-terminal cluster
5	RAMP	COG1688, COG1769, COG1583, COG1567, COG1336, COG1367, COG1604, COG1337, COG1332, COG5551, BH0337-like, MJ0978-like, YgcH-like, y1726-like, y1727-like	All	Belong to "RAMP" superfamily, possibly RNA-binding protein, structurally related to a duplicated ferredoxin fold (PDB: 1WJ9)
6	COG1857	COG1857, COG3649, YgcJ-like, y1725-like	All	α/β protein; probable enzymatic activity, possibly, a nuclease
7	HD-like nuclease	COG1203 (N-terminus), COG2254	All	HD-like nuclease
8	BH0338	BH0338-like MTH1090-like	All, mostly archaea and FIRM	Large Zn-finger-containing proteins, possibly, nucleases (nuclease activity has been reported for MTH1090 [75].
9	ygcL	ygcL	Bacteria, mostly PROTEO	Large Zn-finger containing proteins;
10	COG1353	COG1353, MTH326-like, alr1562, slr7011	All, mostly Archaea	Putative novel polymerase; Multidomain protein with permuted HD nuclease domain, palm domain, polymerase-thumb-like domain and Zn-ribbon; MTH326-like has inactivated polymerase catalytic domain; alr1562 and slr7011 – predicted only on the basis of size, presence of HD domain, and location with RAMPs in one operon
11	COG1517/HTH	COG2462	Archaea	Former COG2462; Fusion of COG1517-like domain to HTH-type transcriptional regulator; Possible regulator of the system expression in archaea
12	COG1421	COG1421	All, mostly Archaea	~150 aa protein; Has a few motifs similar to ygcK-like; mostly α-helical protein
13	ygcK	ygcK-like	Bacteria, mostly PROTEO	~180 aa protein; has a few motifs similar to COG1421; mostly α-helical protein
14	COG3337	COG3337	All, mostly Archaea	~110 aa; mostly α-helical protein
15	COG1517	COG1517 COG4006	All, mostly Archaea	Some are fused to HTH domain (see COG1517/HTH), some proteins have the domain duplication; structure is available (1XMX); domain appears to have a Rossmann-like fold.
16	COG3513	COG3513	Bacteria, mostly PROTEO	Huge protein; contains McrA/HNH-nuclease related domain and RuvC-like nuclease domain
17	PH0918	PH0918-like	All, mostly Archaea	Specific for *Pyrococcus *and *Thermococcus*. The pair ST0031/SSO1401 and AF1873, most likely, belong to the same family because have similar length and located in the identical place in an operon but due to low conservation are not alignable
18	AF1870	AF1870-like	Archaea	Former COG3574; ~150 aa protein.
19	AF0070	AF0070-like	Archaea	~420 aa protein, no prediction
20	y1724	y1724-like	Bacteria, mostly PROTEO	~450 aa protein, no prediction
21	SPy1049	Spy1049-like	Bacteria, mostly FIRM	~220 aa protein, no prediction
22	TTE2665	TTE2665-like	Bacteria, mostly CHLOR	~130 aa protein, no prediction
23	LA3191	LA3191-like	Few bacteria	~650 aa, no prediction

^A ^– Subfamilies are named by the corresponding COG number or by a protein ID ^B ^– All indicates that the family is widespread in all major prokaryotic lineages; PROTEO – proteobacteria; FIRM – firmicutes; CHLOR – Chlorobia

#### COG1343 and its relatives – distant homologs of VapD

COG1343 (cas2) is another gene that is common in CASS. Typically, this gene is located immediately downstream of the COG1518 gene (Fig. 2a). Exhaustive PSI-BLAST search starting from COG1343 proteins identified proteins of COG3512 as homologs of COG1343 such that these COGs could be unified in a single superfamily. The members of this superfamily are small (80–120 amino acids) proteins with distinct structural motifs, in particular, an N-terminal β-strand followed by a polar amino acid, most often, aspartate or asparagine (see Additional file 2). In CASS2, which is typified by the *cas *operon of *E. coli*, there is an uncharacterized gene coding for a small protein immediately downstream of the COG1518 gene. Analysis of the multiple alignment of the homologs of this protein revealed motifs highly similar to those in COG1343, suggesting that these proteins actually are diverged COG1343 homologs. Only CASS3 does not seem to contain a gene for a small protein that potentially could be a COG1343 homolog. However, we found that, in CASS3, the next gene after COG1518, which codes for the CASS helicase (cas3 or COG1203) is unusually long and contains a small domain preceding the HD-hydrolase N-terminal domain present in many COG1203 proteins. We analyzed this domain separately and found that its size and motifs were consistent with a homologous relationship with COG1343 (see Additional file 2). Thus, it appears that, either as a stand-alone protein or as part of a multidomain protein, the COG1343 domain is present in all CASS (except for a few highly degraded and, probably, non-functional ones) and, accordingly, could be essential for the CASS functions. Furthermore, searches started with the sequences of many proteins of COG3512 showed some sequence similarity to vapD (COG3309 family), a family of uncharacterized proteins that are functionally linked to the VapBC operon [34]. The VapBC operon encodes a variant of the bacterial toxin-antitoxin (TA) module which includes an HTH-containing transcription regulator and a PIN-domain nuclease. The PIN domain has been shown to possess ribonuclease activity that in eukaryotes is involved in pre-rRNA processing, nonsense-mediated decay (NMD) of aberrant mRNAs, and RNAi [35-37]. It has been proposed that there is an evolutionary connection between eukaryotic NMD and bacterial TA systems, and that the functioning of the TA module might involve mRNA degradation as well [38].Together, these observations seem to establish links between CASS and the TA system and, through the latter, between CASS and eukaryotic NMD and RNAi, but do not directly shed light on the function of the COG1343 domain. However, the COG1343 proteins and VapD show some generic similarity to the PIN domain in terms of size and the signature motifs (COG1343 proteins contain several partially conserved aspartates; see Additional file 2) which makes it tempting to speculate that these proteins represent yet another family of nucleases.

#### COG1857 – another putative enzyme found in most CASS versions

**Figure 3:**
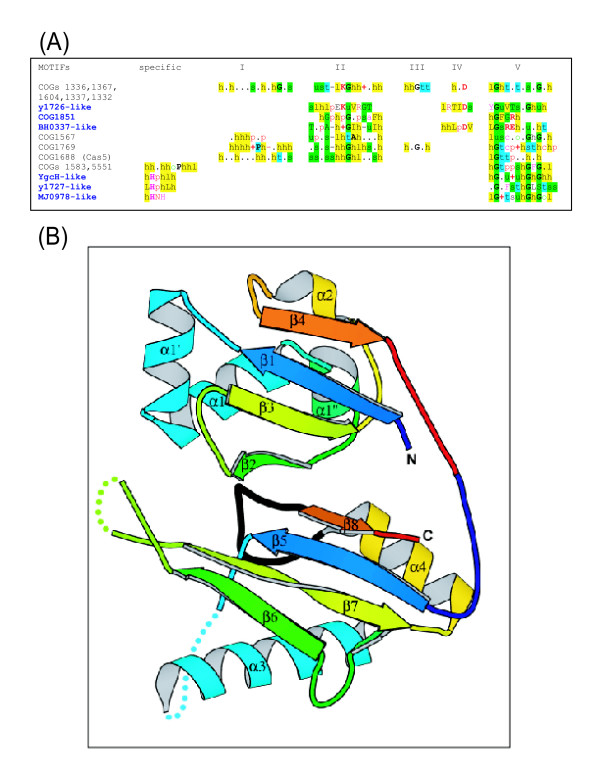
**The RAMPs**. (A) The conserved motifs of the RAMP superfamily and individual RAMP families. h designates a hydrophobic residue, p designates a polar residues, t designates a residue with high turn-forming propensity, and + designates a positively charged residue. (B) A ribbon model of the structure of a RAMP protein from *Thermus thermophilus *(PDB entry 1wj9). Two ferredoxin-like domains are rainbow-colored from N- to C-terminus such that the corresponding strands in the two each domain receive the same color. The G-rich conserved loop in the C-terminal domain is colored black, structurally disordered regions are shown by dots, α-helices and β-strands are numbered consecutively throughout the sequence from α1 to α4 and from β1 to β8.

Another conserved CASS family that resists straightforward functional prediction is COG1857. This gene is present in most CASS, with the exception of the "minimal" variants, CASS 4, 6, and 7. The COG1857 proteins consist of ~350 aa and typically have an N-terminal β-strand followed by a loop containing a conserved asparagine (see Additional file 3). Although no other motifs containing potential catalytic residues seem to be conserved throughout COG1857, perhaps, due to the extensive divergence between the subfamilies of this family, the observed conservation pattern suggests that this protein is an enzyme, perhaps, yet another nuclease. COG1857 proteins are typically encoded immediately upstream of another widespread CASS gene, COG1688 (named Cas5 by Haft et al. [24]). We have previously identified COG1688 as a member of the Repair-Associated Mysterious Protein (RAMP) superfamily [23] that has been renamed to Repeat-Associated Mysterious Protein (preserving the acronym) by Haft and coworkers [24]. In CASS3, COG1688 is replaced by the y1726 family which we found to include another set of RAMPs distantly related to COG1688 (Figs. 2a and 3A).

#### The Repeat-Associated Mysterious Proteins (RAMPs)

The RAMPs are the most diverse class of CASS genes. In addition to the previously identified 5 distinct families of RAMPs, we detected several additional ones, namely, BH0337-like, y1726-like, YgcH-like, y1727-like, and MJ0978-like families, as well as numerous diverged members of the previously described families (Fig. 3A). Despite the dramatic sequence divergence, all these protein contain the RAMP signature, the G-rich loop at the C-terminus (Fig. 3A). One family of RAMPs, COG1853/COG5551, is often encoded outside the CASS operons or on the periphery of these. Moreover, analysis of the gene context of this gene in *Aquifex aeolicus *led us to the identification of yet another member of RAMP superfamily, COG1851. This protein family does not appear to be linked to CASS at all.

With the identification of these new families of RAMPs, it now becomes apparent that all CASS versions, with the apparent exception of the minimal CASS4, include at least one RAMP. The crystal structure of one of the RAMPs from the newly detected YgcH-like family has been solved as part of one of the structural-genomics projects (PDB: 1wj9). The structure of this protein from *Thermus thermophilus *reveals that the RAMP module is a duplication of a ferredoxin-like fold domain. Each domain has a two-layer α+β architecture and is composed of four β-strands and two α-helices topologically arranged as a repeat of two βαβ units (Fig 3B, A1-A86, β1 through β4; and A87-A211, β5 through β8 for the first and the second domains, respectively). The N-terminal ferredoxin-like domain contains two additional α-helices (α1' and α1", Fig 3A) inserted before and after the first α-helix. The C-terminal domain has two disordered regions and houses the conserved Gly-rich loop situated between the last α-helix and β-strand (Fig. 3A). Various structure similarity search programs detect ferredoxin fold proteins as the first hits to RAMP domains. In particular, for the N-terminal RAMP domain, DALI [39], first match is the anticodon-binding domain of Phe-tRNA synthetase (PDB Entry 1eiy, chain B) with Z-score 4.8 and RMSD (squared Root of Mean Square Deviations) 2.9Å over 67 aligned residues. The VAST program [40] finds ribosomal protein S6 (PDB Entry 1fjg chain F) as the top hit for the C-terminal RMAP domain with P-value 0.039, RMSD 2.6 Å over 64 residues.

Thus, at least six gene (super)families seem to comprise the stable core of the CASS: COG1518 (*cas1*), COG1343 (*cas2*), COG1203 – a helicase often fused to a HD-family hydrolase (*cas3*) plus free-standing versions of the HD-hydrolase (COG2254), COG1468 (*cas4*) – a RecB family nuclease usually containing a C-terminal Zn cluster, COG1857, and RAMPs. This exact set of genes is seen only in a few genomes; most versions of CASS have substantial variations around this core – loss of some core genes in the minimal versions and addition of other genes and whole gene cassettes in others (Fig. 2a,b).

#### The second major module of CASS – the pol-cassette

The most notable non-core CASS module which, in a sense, constitutes a second, even if non-ubiquitous, central part of CASS may be called the "pol-cassette" after the predicted palm-domain RNA or DNA polymerase of COG1353. The pol-cassette also includes several distinct RAMPs and a few uncharacterized genes. The pol-cassette is strictly linked only to CASS5 and CASS7 and also, in some instances, is found in CASS1,2,4,6 (Fig. 2c), although, in some genomes, the pol-cassette is not adjacent to the CASS-core gene array. The phylogenetic tree for the predicted polymerase (COG1353), which consisted of two major branches corresponding to two distinct operon organizations (Fig. 2c), showed essentially no topological congruence with the COG1518 tree (for those CASS that have both components; compare the two trees in Fig. 2a,c). Thus, it appears that the pol-cassette comprises a distinct evolutionary unit that is often transferred horizontally independently of the CASS-core. Notably, the pol-cassette is strongly, although not strictly, linked to thermophily – the great majority of the species containing this module, typically associated with CASS5 and CASS7, are thermophiles. Additionally, several species possess a third module containing a diverged form of COG1353 with an apparently intact HD hydrolase domain but an inactivated polymerase (PALM) domain (Fig 2c).

#### Ancillary CASS components

**Figure 4:**
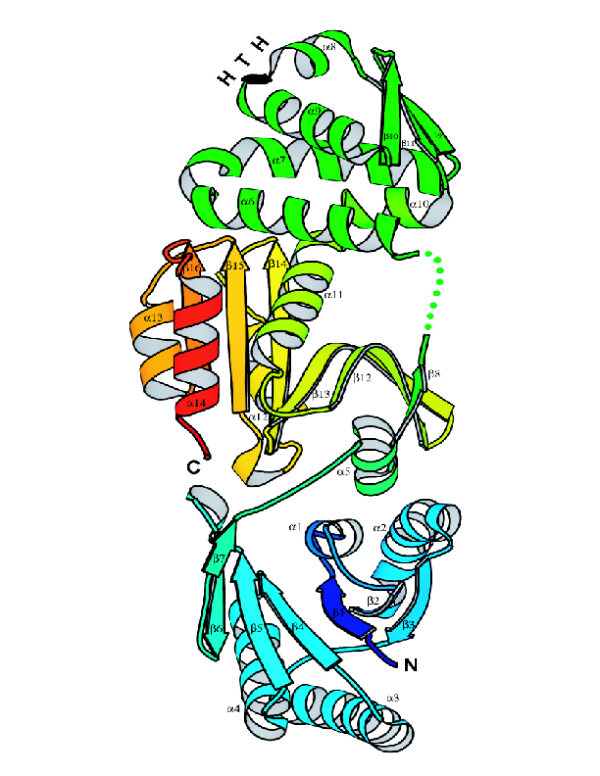
**A ribbon model for the structure of a COG1517 protein, Vc1899 from *Vibrio cholerae *(PDB entry **1xmx). The structure is rainbow-colored from N- to C-terminus such that each of the three domains is assigned a visually distinct region of the color spectrum: blue, the modified Rossmann-like fold; green, the winged helix-turn helix (HTH) domain; yellow-orange, the endonuclease-like domain. The T-turn in the HTH is colored black, a structurally disordered region is shown by dots, α-helices and β-strands are numbered consecutively throughout the sequence from α1 to α14 and from β1 to β16.

The CASS-core and the pol-cassette together comprise the extended central componentry of CASS that consists of six COGs plus the RAMP superfamily (Table 1). In addition, ~20 other families were consistently found in CASS, even if in a minority of the CASS-containing species (Table 1). It has to be taken into account that most of the CASS proteins have highly diverged sequences, and further unification as well as expansion and the ensuing rise in status of some of the families remains a possibility. For example, COG1517 is such a growing family. Typically, the COG1517 genes are located on the periphery of pol-cassettes and, in many cases, contain fused helix-turn-helix (HTH) domains. The core domain of COG1517 is ~150 aa; several proteins, e.g. TM1812, contain a duplication of this domain, and several fusions, in addition to the one with HTH, were detected. The sequences of COG1517 proteins tend to be highly diverged, and some of these were detected in genomes with no CASS, indicating that the association of this gene with CASS is not as tight as it is for the extended core of CASS. For one such protein from *Vibrio cholerae*, the 3D structure has been solved, again, in one of the structural genomics projects (PDB: 1xmx). The structure is composed of three domains. The N-terminal domain (A-1-A137, β1 through β7, Fig. 4) is a modified Rossmann-like fold, with the next to the last β-strand (β6) being antiparallel to the rest of the strands. The middle domain (A161-A249, α6 through α10) is a winged helix-turn-helix (HTH) with an additional α-helix on each terminus (α6 and α10, respectively). The C-terminal domain (composite of A250-A383 and A138-A156, α11 through α14 plus α5 and β8) belongs to the restriction endonuclease superfamily. In the 1xmx structure, the endonuclease domain is positioned between the HTH and the Rossmann-like domain (Fig. 4). The lowest VAST P-values of the matches between each of the 1xmx domains and the respective domains with known folds are highly statistically significant (<10^-5^) which is strongly suggestive of homologous relationships.

The functions of several other CASS gene families remain obscure. For instance, CASS1, 5, 7 contain genes upstream of COG1857 that encode large (500–600 amino acids), homologous proteins; the best conserved family in this set of proteins is represented by BH0338 and its orthologs. Some members of the BH0338 family contain a Zn-ribbon in the middle of the sequence but otherwise have no recognizable domains or motifs. Among several conserved motifs of these proteins, are two conserved aspartates and a distal conserved glycine, a combination that resembles the motifs seen in the PALM polymerase domain (not shown). Although we were unable to obtain additional evidence of the potential connection of this family with polymerases, it is tempting to speculate that these proteins might contain an extremely diverged version of the PALM domain. Similar, albeit less pronounced, motifs are detectable in the MTH1090 family proteins which are present in CASS5 and CASS7. This similarity and the fact that the respective genes occupy the same position in the corresponding operons suggest that the BH0338-family and the MTH1090-family proteins are highly diverged homologs. CASS2 also includes a large protein (YgcL-family), some of which contain Zn-clusters; however, the conserved motifs of these proteins and their position in the respective operons are different from those of the MTH1090 and BH0338 families. CASS4 contains another huge protein (COG3513, ~1150–1400 aa) with two recognizable domains, a McrA/HNH nuclease and a RuvC-like nuclease (RNAseH fold). These observations emphasize the striking diversity of still poorly characterized CASS components, particularly, the plethora of predicted nucleases of various classes and potential novel ones.

### Hypothesis: CASS is a prokaryotic defense system that functions on the RNAi principle

Based on the properties of CRISPR and Cas proteins, we speculate that this system is a functional analog of the eukaryotic siRNA systems and propose possible mechanisms of the putative prokaryotic small RNA interference. The crucial observation reported independently by Mojica et al [27], Pourcel et al [29], and Bolotin et al [28] is that a certain fraction (~10% according to [27]) of the unique inserts in CRISPR units are homologous to fragments of viral (bacteriophage) or plasmid genomes. Only a miniscule fraction of the existing phage and plasmid sequences is currently available, whereas the total diversity of prokaryotic mobile elements is humungous and apparently exceeds the diversity of prokaryotes at least by an order of magnitude [41,42]. Thus, it is not far fetched to propose that most, if not all, CRISPR inserts are derived from mobile replicons [29].

Should that be the case, it seems more or less obvious that CASS is a prokaryotic defense system against foreign replicons that functions on the antisense RNA principle. More specifically, it seems likely that the inserts are transcribed and silence the cognate phage or plasmid genes via the formation of a duplex between the prokaryotic small interfering (psiRNA) and the target mRNA followed by cleavage of the duplex or translation repression. Indeed, Mojica et al [27] mention the analogy between the CRISPR and eukaryotic RNA interference systems but propose no specific mechanisms for the action of the putative defense systems and, crucially, do not explore the connection between the putative psiRNA and the predicted activities of Cas proteins. Important supporting evidence has been independently obtained through the analysis of the small non-messenger RNA expression in the euryarchaeon *Archaeoglobus fulgidus *which showed that CRISPR are transcribed (from a leader-promoter sequence), apparently, in the form of a multiunit precursor that is subsequently cleaved into CRISPR monomers and oligomers [18]; very similar observations have been subsequently reported for the crenarchaeon *Sulfolobus solfataricus *[19]. Furthermore, as noticed by Pourcel and coworkers [29], one of the unique CRISPR inserts in the MIGAS strain of the bacterium *Streptococcus pyogenes *is homologous to a prophage present in other strains of the same bacterium that, conversely, do not carry the CRISPR. This is compatible with the possibility that the insert makes the bacterium immune to the given phage.

Here, we pursue these lines in an attempt to present a coherent, even if speculative, description of the putative prokaryotic siRNA system. Circumstantial but crucial evidence in support of the psiRNA hypothesis comes from analogies between the predicted functions of CASS proteins and the protein components of eukaryotic RNA interference systems, in particular, the RISCs [7-9] (Table 2). The core parts of RISCs are a helicase fused with two RNAse III domains, dicer, and the exonuclease of the argonaute family, slicer [9,10,43]. Both dicer and slicer are represented by variable numbers of paralogs in eukaryotes, and different paralogs are included into RISCs with distinct functions [9,10]. Various RISCs also contain additional RNA-binding proteins and nucleases [9,44]. Putative functional analogs of all these proteins can be gleaned among the CASS proteins. The dicer analog is immediately apparent: the COG1203 helicase that is either fused or encoded next to a predicted nuclease of the HD family (Fig. 2a); we tentatively designate this protein p-dicer (prokaryotic dicer). Identification of the slicer counterpart (p-slicer) is less straightforward because of the diversity of predicted nucleases within the CASS. One candidate is the predicted RecB-family nuclease (COG1468). Alternatively or additionally, the slicer function could be performed by a novel, still unidentified nuclease, such as COG1857. Indeed, the possibility cannot be dismissed that different or even the same version of CASS employ multiple p-slicers, in a parallel with the multiple, paralogous eukaryotic slicers of the argonaute family. We further propose that RAMP proteins, by far the most diverse components of the CASS, play a major role in the prokaryotic siRNA mechanism as RNA-binding proteins that display a degree of specificity to the psiRNAs, most likely, by specifically binding psiRNAs of different sizes. The RAMPs, diverse representatives of the same protein superfamily, can be functional analogs of the more structurally diverse RNA-binding proteins of eukaryotic RISCs (Table 2). The presence of two ferredoxin-fold domains in RAMPs (Fig. 3B) is compatible with this proposal given that this fold is seen in a broad variety of RNA-binding proteins, such as the ribosomal proteins S6 and S10, several spliceosomal subunits, and others. Although the structural similarities between the two domains of RAMP and ferredoxin-like domains are not particularly strong, it might be significant that the top structural neighbors for each of the RAMP domains are different RNA-binding domains (see above).

**Table 2:** Functional and structural parallels between CASS and eukaryotic RNAi machinery

**Eukaryotic RNAi**	**Domains/function**	**CASS**	**Domains/function**
Dicers	Helicase/RNAseIII. Processing of long dsRNA into siRNA and pre-miRNA into miRNA, involves unwinding	Helicase (COG1203) + HD nuclease (COG2254) - fused or adjacent genes,	SFII helicase + HD nuclease
Argonautes/slicers	Ferredoxin-fold-PAZ-PIWI – endonuclease, target degradation	RecB-family nuclease (COG1468, 4343); COG1857 – a novel nuclease?	Target degradation
R2D2/RDE-4	dsRNA-binding domain, interacts with Dicer	RAMPs	Ferredoxin-fold duplication. Size-specific psiRNA-binding, pre-psiRNA-binding, other RNA-binding functions?
Fmr1/Fxr	RGG, KH-ssRNA-binding	RAMPs	Ferredoxin-fold duplication. Size-specific psiRNA-binding, pre-psiRNA-binding, other RNA-binding functions?
Tsn	Tudor, SN – RNA-binding	RAMPs	Ferredoxin-fold duplication. Size-specific psiRNA-binding, pre-psiRNA-binding, other RNA-binding functions?
Vig	RGG – RNA-binding	RAMPs	Ferredoxin-fold duplication. Size-specific psiRNA-binding, pre-psiRNA-binding, other RNA-binding functions?
RNA-dependent RNA polymerase	RdRp domain related to DdRp; 2^nd^-strand synthesis for siRNA production	Predicted RdRp/RT (COG1353)	Palm polymerase domain. 2^nd ^strand synthesis for psiRNA production, reverse transcription for CRISPR formation

**Figure 5:**
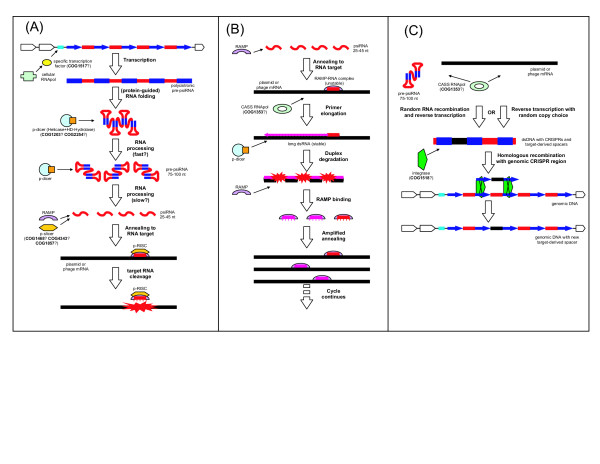
**The current hypothetical model for CASS functioning and CRISPR formation**. (A) The basic model of CASS functioning (B) The variant of CASS functioning involving the CASS polymerase (C) Formation of new CRISPR with unique inserts.

Figure 5a presents our current hypothesis on the basic mechanism of the functioning of the prokaryotic RNAi system. We speculate that CRISPR regions are transcribed from a promoter located in the AT-rich CRISPR leader. Transcription might be regulated with the participation of one of the Cas proteins and, conceivably, would be stimulated by stress, such as phage infection, although the results of expression analysis in *A. fulgidus *suggest some level of constitutive transcription [18]. The work of Tang and coworkers further indicates that the primary transcript is likely to encompass the entire CRISPR repeat region. This transcript would be cleaved into 70–100 nt pre-psiRNA, conceivably, by the putative p-dicer, the COG1203 protein (Fig. 5a). We further postulate that p-dicer catalyzes the second, perhaps, slower (judging by the results of Tang et al) processing step that releases mature psiRNA species (Fig. 5a). The psiRNA molecules would bind RAMPs in a size-specific manner and anneal to the target mRNA. The resulting complex would recruit p-slicer, forming the minimal form of the prokaryotic analog of RISC (pRISC) that would cleave the mRNA and could be recycled to attack the next target molecule, thus, silencing the respective gene (Fig. 5a).

Figure 5b shows a version of this pathway that involves the activity of the CASS polymerase, by analogy with the eukaryotic RdRp, which participates in some RNAi pathways in most eukaryotes, but apparently has been lost in arthropods and chordates [45-47]. The initial steps in this scheme are the same as in the basic one (Fig. 5a) – transcription of the CRISPR, processing of the psiRNA precursor, and annealing of psiRNA to the target mRNA mediated by the pRISC – but, at the next step, psiRNA is postulated to serve as the primer for elongation by the CASS polymerase, yielding an extended double-stranded form of the target (Fig. 5b). This form would be cleaved by p-dicer analogously to the cleavage of viral and transposon dsRNAs by the eukaryotic dicers. The p-dicer might function as a complex with the respective RAMP to form a distinct version of pRISC. This could be the endpoint of the pathway, or else, the dsRNA degradation products could be utilized as new psiRNAs, resulting in amplification of the silencing effect (Fig. 5b). The CASS polymerase is most common in thermophiles, and it is tempting to speculate that the prevalence of this form of the psiRNA pathway has to do with the instability of the psiRNA-target duplex under the high ambient temperatures of these organisms.

The most complex and uncertain aspect of the putative prokaryotic RNAi system discussed here is the formation of new CRISPR units containing unique psiRNA genes specific for new targets encountered by the organism (Fig. 5c). The path to the creation of new psiRNAs would begin just like the response pathway, i.e., with transcription of the CRISPR locus and the first processing step yielding the 70–100 nt psiRNA precursors (compare Fig. 5c with Fig. 5a). At the next step, however, there must be a mechanism to replace the unique insert within the pre-psiRNA with a new fragment of foreign (e.g., phage) RNA. The nature of this mechanism remains unclear. In principle, two possibilities can be envisaged: i) reverse transcription with copy choice whereby a reverse transcriptase, most likely, the predicted CASS polymerase (COG1353) switches from using the pre-psiRNA as a template to using a phage mRNA, and then back, and ii) direct, non-homologous RNA recombination between a pre-psiRNA and a foreign mRNA, followed by reverse transcription of the resulting recombinant RNA (Fig. 5c). Both mechanisms are non-trivial in their molecular choreography and are unlikely to occur with high efficiency. Nevertheless, there are precedents for both in the molecular biology of retroviruses and other RNA viruses. In particular, reverse transcriptase switches templates in each cycle of retrovirus first-strand cDNA synthesis although, in this case, copy-choice is facilitated by the spatial juxtaposition of the two templates within the virus particle; a similar mechanism is responsible for recombination in retroviruses [48]. In addition, and probably, more relevantly to the psiRNA case, reverse transcription with copy-choice is thought to be involved in the incorporation of copies of cellular genes, such as oncogenes, into retroviral genomes [49,50]. The alternative, namely, direct recombination between RNA molecules might seem far fetched, but such a process has been demonstrated, by several groups independently, to occur in RNA viruses, apparently, via a protein-independent mechanism [51,52]. During the formation of new psiRNA species, these low-frequency processes might be facilitated by the high abundance of the phage mRNAs involved. Indeed, it has been shown that the unique inserts in CRISPR most often correspond to fragments of essential, highly conserved phage genes that are typically expressed at a high level in infected bacteria[27]. Once the dsDNA molecule consisting of a CRISPR unit with the new, unique insert is produced, by one of the mechanisms outlined here or, perhaps, via a different pathway, it must insert into the CRISPR array via homologous recombination (Fig. 5c). We suspect that this process is mediated by the COG1518 protein, the universal marker of CASS containing conserved motifs resembling those of different nucleases [23]. It seems likely that this protein functions as the CRISPR integrase/recombinase, perhaps, in cooperation with the COG1343 protein, another universal component of CASS.

### Additional lines of evidence relevant for the predicted RNAi function of CASS

#### Genes loosely associated with CASS

In addition to the bona fide (even if not ubiquitous) CASS components, a variety of genes were found in association with CRISPR and CASS in only one or a few closely related genomes. Thus, functional association of these genes with CASS appears uncertain. However, examination of the list of such genes clearly indicates that they are not a random set (Table 3). Similarly to the common CASS components, there are several nucleases on this list. More notable is the presence of the reverse transcriptase (RT) which, on at least two independent occasions, has been fused to the COG1518 gene (Fig. 2a and Table 3). This fusion suggests the intriguing possibility that, in the respective variants of CASS, the RT (probably derived from retron-type elements) takes over the proposed function of the CASS polymerase (Fig. 5c); this appears to provide indirect but substantial support for the reverse-transcription-mediated mechanism of CASS function. Perhaps, the most remarkable observation is the presence of the archaeal homolog of the eukaryotic argonaute protein (the slicer) in one of the CASS operons of the archaeon *Methanopyrus kandleri *(Table 3). This suggests the possibility of a functional association of this protein with CASS, at least in some archaea, and provides the only putative (even if weak – until other archaeal genomes with such a gene arrangement are found) link between CASS and the eukaryotic RISCs at the level of homologous proteins.

**Table 3:** Genes loosely associated with CASS

	**Family**	**Example of genes associated with CASS**	**Comments**
1	Reverse transriptase (RT)	VVA1544, PG1982, alr1468	Fused to COG1518 in three occasions and a remnant of RT (Mbar_A1351 and MM3360) in *M. barkeri *and *mazei *genomes is located close to cas
2	PIN-domain	alr1560, ST0017, Ava_4168	Ribonuclease
3	COG2442	Ava_4167	HTH domain, component of toxin-antitoxin system, probably targeting mRNA
4	COG1432	MS0983	Large family of proteins, predicted to be a phosphatase or a nuclease on the basis of sequence motifs which is shared by all three domain of life. In multidomain proteins in plants it is associated with C2H2 Zn-finger domain
5	PA2117-like	MS0982, MS0989	An enzymatic domain, that is located in an operon with restriction-modification systems or in association with a diverged helicase
6	COG3645-like	ACIAD2479	Homologs of phage anti-repressor Ant which is known to be inhibited by an antisense RNA
7	argonaute	MK1311	Homolog of the eukaryotic argonaute protein, that are key player in RNA guided posttranscriptional regulation by siRNA and miRNA
8	COG1598/COG4226/HicB	MCA0653, MTH321	Probably has an RNAseH-like fold, often fused to CopG-family of transcriptional regulators; forms a conserved operon with COG1724/hicA, which has the dsRBD-like fold; possible novel toxin-antitoxin module targeting mRNA
9	PUA-domain	LIC10933	RNA binding domain
10	3'-5' exonuclease	LcasA01001274	Fused to COG1343 in *Lactococcus bulgaricus *and *L.casei*
11	COG1652	TK0459	Regulatory ATPase of AAA family fused to RecB-family nuclease; Predicted regulator of RNA metabolism
12	AbrB/MazE domain	TK0457, PAE0118	DNA-binding domain, belongs to the same fold as MazE, which involved in toxin-antitoxin system
13	S1-domain	CaurDRAFT_2121	Ribosomal protein S1-like RNA-binding domain, fused to RAMP domain
14	CSP-like	Rrub02003211	Cold shock protein-like RNA-binding domain, fused to RAMP domain

#### A connection between CRISPR and RAMPs

**Figure 6:**
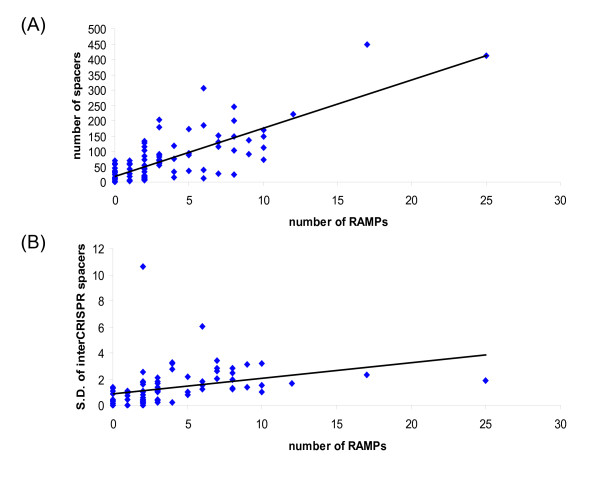
**CRISPR and RAMPs**. (A) Correlation between the number of encoded RAMPs and the number of CRISPR units in prokaryotic genomes (B) Correlation between the number of encoded RAMPs and the variance of unique insert lengths of CRISPR-related spacers in prokaryotic genomes.

We searched for CRISPR in the 89 complete prokaryotic genomes containing cas genes and found remarkable diversity, in both the number of repeat units and the spacer size, even among closely related species and strains, which is in agreement with previous observations [25,27-29]. Given our hypothesis that RAMPs are size-specific psiRNA-binding proteins, we examined possible connections between the number of RAMP genes, the number of CRISPR units (which is the same as the number of unique inserts) and the length heterogeneity of the unique inserts in prokaryotic genomes. The number of RAMPs showed strong positive correlations both with the number of CRISPR units (Fig. 6a) and with the variance of the insert lengths (Fig. 6b) which seems to be compatible with our hypothesis on the psiRNA-binding function of RAMPs. Significant correlations were observed also between the number of CRISPR and the variance of the insert length, and between each of these variables and the number of COG1518 proteins, COG1517 proteins or all Cas proteins minus the RAMPs. However, the correlations seen for the RAMPs and the CRISPR inserts were the strongest, suggesting that these were the biologically most relevant links (Table 4). It should be emphasized that, given the extreme sequence divergence of the RAMPs, it is most likely that the correlation between the number of encoded RAMPs and the number of CRISPR units present in a genome is due to the RAMPs discriminating between inserts (by size, under our hypothesis) rather than recognition of the repeats themselves.

**Table 4:** Rank correlations coefficients between CRISPR spacers and selected Cas proteins

Values selected for correlation	Rank Correlation coefficient^a^
Number of spacers vs S.D. of spacer lengths	**0.398**
Number of spacers vs number of COG1518 proteins	**0.475**
Number of spacers vs number of RAMPs	**0.668**
Number of spacers vs number of COG1517 proteins	**0.524**
Number of spacers vs total number of Cas proteins minus RAMPs	**0.580**
Number of spacers vs total number of Cas proteins minus RAMPs and COG1517 proteins	**0.556**
Number of RAMPs vs S.D. of spacer lengths	**0.651**

^a^All correlation coefficients are highly statistically significant P < 10^-3^

### Putative psiRNAs: relationships with phage and plasmid genes and secondary structure

We sought to reassess the relationship between the unique CRISPR inserts and bacteriophage and plasmid genes with the currently available, increased collection of prokaryotic and phage genomes. Using somewhat more stringent criteria than those adopted by Mojica et al. [27], we found that only a small fraction the unique CRISPR inserts showed significant similarity to any sequences in the current databases. Altogether, we identified 83 inserts with apparent homologs, or ~2% of all available CRISPR sequences. Importantly, the great majority of these inserts were indeed homologous to fragments of phage or plasmid genes rather than to sequences from bacterial or archaeal chromosomes (Table 5). Rather unexpectedly, it turned out that CRISPR inserts homologous to phage and plasmid genes came in both the sense and the antisense orientations (Table 5). Although determination of the direction of transcription for CRISPR cassettes is ambiguous because the leader sequence thought to include the CRISPR promoter is hard to identify, it is certain that some of the CRISPR cassettes contain inserts of both orientations (Table 5). This finding presents a complication to the schemes of CRISPR functioning presented in Fig. 5 because sense fragments of genes, obviously cannot silence the respective targets directly. There seem to be three possible solutions to this problem, all based on the assumption that the recombination process in the scheme shown in Fig. 5c is random with respect to direction. The first possibility is that the sense inserts are non-functional by-products of indiscriminate recombination. Alternatively, it is conceivable that both sense and antisense inserts are converted to duplexes, probably, by the CASS polymerase, after which the sense strand is destroyed whereas the antisense strand becomes part of the pRISC. A very similar mechanism involving the RNA-dependent RNA polymerase has been described for one of the endogenous siRNA pathways in plants [53]. The third, more exotic possibility is that psiRNA actually function not by silencing mRNAs but by promoting degradation of the target DNA, in which case both sense and antisense psiRNAs could be active. This option is further discussed in the Conclusions section.

**Table 5:** A selection of CRISPR inserts homologous to phage, plasmid and prokaryotic genes

Species	Total spacers	Phage Plasmid	sense	antisense	Other prokaryotes.
*Sulfolobus solfataricus*	413	22	9	12	4
*Sulfolobus acidocaldarius *DSM 639	222	0			1
*Sulfolobus tokodaii*	449	4	4	0	2
*Xanthomonas oryzae *KACC10331	59	14	13	1	0
*Anabaena variabilis *ATCC 29413	174	0			1
*Methanobacterium thermoautotrophicum*	169	10	4	4	0
*Methanocaldococcus jannaschii*	185	0			1
*Bacillus halodurans*	89	3	1	1	0
*Streptococcus thermophilus *CNRZ1066	41	20	15	5	0
*Streptococcus thermophilus *LMG 18311	37	10	5	5	0
*Streptococcus agalactiae *2603	24	0			1

Importantly, the CRISPR insert sequences from even closely related bacterial strains are unrelated to each other, with only a few exceptions among enterobacterial strains (data not shown). This suggests that the inserts are replaced rapidly on the evolutionary scale, perhaps, via the mechanism outlined in Fig. 5c. A broader implication is that the dominant phages and plasmids encountered by even closely related bacteria are different leading to the rapid generation of distinct repertoires of psiRNAs.

**Figure 7:**
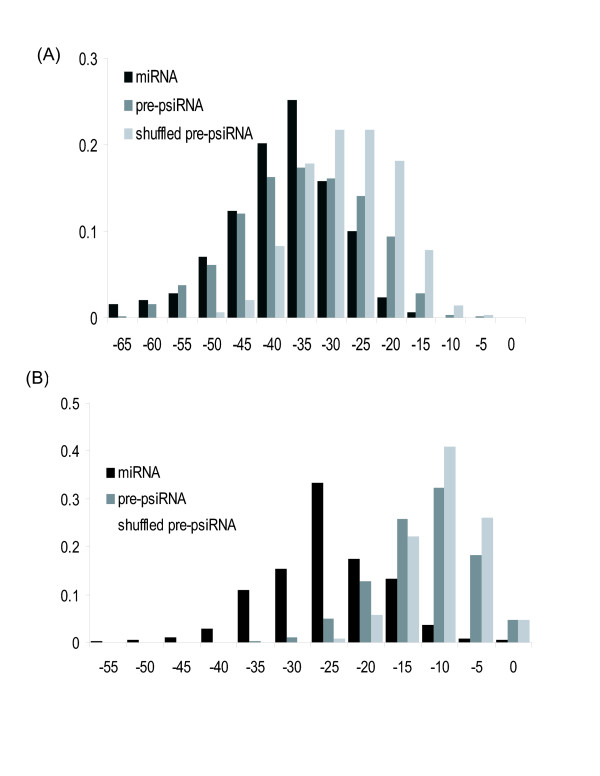
**Folding free energy distributions for the putative psiRNA precursors**. (a) GC-rich psiRNA precursors compared to the corresponding shuffled sequences and miRNAs (b) AT-rich psiRNA precursors compared to the corresponding shuffled sequences and mRNAs. The X-axis: folding energy.

In terms of their ultimate function, the putative psiRNAs appear to be conceptually most similar to eukaryotic siRNAs in that they are homologous to foreign, rather than endogenous, genes and are predicted to function as part of a defense system. However, the psiRNAs also share a major feature with the miRNAs in that they are embedded in and, probably, cleaved from a specific precursor molecule, rather than from a long dsRNA like the siRNAs (Fig. 5). In an attempt to elucidate common features of the psiRNA precursors, we predicted the secondary structures of all available CRISPR units. The results were somewhat ambiguous as shown in Fig. 7. The folding free energies of GC-rich CRISPR units were substantially lower than those for the shuffled CRISPR units; notably, the CRISPR folding energies were distributed similarly to those of miRNA precursors (Fig. 7a), with numerous CRISPR units capable of folding into highly stable secondary structures. These observations suggest the possibility that psiRNA precursors possess a distinct secondary structure that could be important for recognition by p-dicer, RAMPs or other proteins. However, the folding energy distribution for AT-rich CRISPR shows only a slight shift compared to the distribution for shuffled sequences and is completely different from the distribution for miRNA precursors of the same base composition (Fig. 7b). Thus, AT-rich psiRNA precursors, which comprise approximately half of the CRISPR, on average, do not seem to be capable of folding into stable secondary structures. Thus, the existence of a functionally relevant consensus structure of psiRNA precursors remains uncertain.

**Figure 8:**
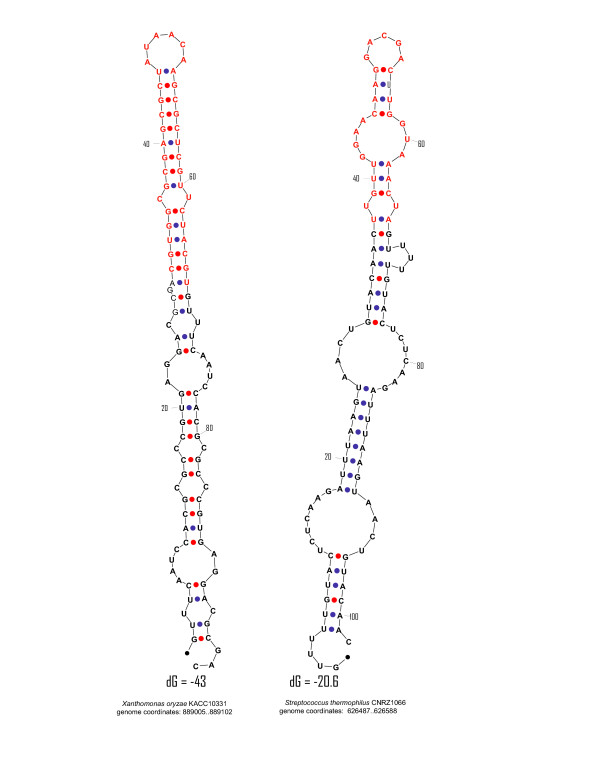
**Two predicted structures of putative psiRNA precursors**. The unique inserts are shown in red, and the CRISPR sequence is shown in boldface.

Figure 8 shows two predicted secondary structures of putative psiRNA precursors in which the inserts are homologous to phage or plasmid genes. The relatively GC-rich sequence from *Xanthomonas axonopodis *forms a stable secondary structure, whereas the AT-rich sequence from *Streptococcus thermophilus *can fold only into a weak stem-loop structure. Curiously, in both cases, the unique insert contributes significantly to stem formation. Clearly, further analysis of the structures of putative psiRNA precursors by computational and experimental means is required to identify their functionally important features.

## Conclusion

Obviously, the entire concept of the prokaryotic CASS defense system functioning on the RNAi principle currently remains a hypothesis. However, we believe that three lines of evidence make such a mechanism, in its broad outline, almost a logical inevitability: i) the indisputable origin of at least some of the unique CRISPR inserts from phage and plasmid genes, ii) the demonstration of transcription and processing of the CRISPR loci in *A. fulgidus *and *S. solfataricus*, and iii) the abundance of CASS components that are clearly implicated in nucleic acid degradation, processing, and possibly, recombination. The only substantial variation on the theme of RNAi could be an antisense mechanism acting on DNA. Such a mechanism is, obviously, much less common than post-transcriptional gene silencing by small RNAs, but is not without precedent. Indeed there are strong indications that elimination of intergenic DNA sequences in the macronucleus of the ciliate *Tetrahymena *occurs via a siRNA-mediated mechanism, with the participation of a specific dicer-slicer pair [54,55]. In principle, this mechanism is compatible with the finding of both sense and antisense sequences among CRISPR inserts homologous to phage and plasmid genes (Table 5). Nevertheless, given the much wider prevalence of silencing pathways and, in particular, the demonstrated instances of antisense RNA regulation in bacteria, it seems most likely that CASS acts by silencing genes from invading replicons. This being said, it should be emphasized that the mechanisms depicted in Figure 5 are only rough outlines of some of the ways in which this system could function. There is no doubt that experimental studies will reveal mechanisms different from these, at least, in detail. However, regardless of the specific mechanisms and even whether the predicted psiRNA systems acts on RNA or on DNA, it appears certain that its main function is RNAi-mediated defense against alien replicons invading archaea and bacteria.

The predicted psiRNA system resembles the eukaryotic counterparts not only in its functional principle but also in the general characteristics of the implicated proteins. What is most striking is the comparable complexity, diversity, and plasticity of the protein machineries involved. Both systems consist of one or more helicases, a broad spectrum of nucleases, a specific polymerase, and a variety of RNA-binding proteins. Both in CASS and in RISCs, only two or three protein subunits appear to be truly indispensable; the rest come and go, resulting in a variety of RISCs with their distinct functions, many of them still poorly understood [7-9], and, presumably, in a comparable diversity of CASS. Remarkably, however, not a single protein belonging to the bona fide CASS has an ortholog in eukaryotes, involved in RNAi or otherwise. The single direct link could be the argonaut protein which is the central active moiety of eukaryotic RISCs (slicer) and might have some functional connection to CASS in archaea as tentatively suggested by the *M. kandleri *CASS operon structure; admittedly, however, the indications of potential involvement of argonaut in the CASS functioning are currently quite weak.

The eukaryotic RNAi systems come in two basic varieties: i) siRNAs that are produced from dsRNAs of viruses and transposons and protect the host from the respective agents via perfect base-pairing with the respective target mRNAs, and ii) miRNA that regulate translation of endogenous genes via either perfect (plants) or imperfect (animals) base-pairing [6]. CASS appears to be the functional counterpart of the siRNA mechanism inasmuch as it seems to be involved in defense against infecting agents, the psiRNAs seem to be derived from the invading genome and are predicted to function via perfect base-pairing with the target. From a different viewpoint, however, this system is similar to miRNA in that the active small RNA moieties are encoded in the prokaryotic genomes rather than produced from the foreign dsRNA. The closest eukaryotic analogs of the CASS system might be the rasiRNAs that, like the putative psiRNAs, are encoded in the genome, are generated by processing of double-stranded molecules formed by symmetric transcripts of transposons (or repeats thought to be of transposon origin) and silence the latter, which contributes to heterochromatin formation [11-13,56]. In contrast, the bacterial small antisense RNA regulatory pathways that employ Hfq and RNAse E that target regular, chromosomal bacterial genes, rather than those of infectious agents or transposons [14-16], seem to be the functional counterpart of eukaryotic miRNA systems. Thus, prokaryotes seem to have at least two distinct RNAi systems none of which is operated by homologs of eukaryotic RNAi protein machinery components. Furthermore, unlike the case of eukaryotes where the siRNA, rasiRNA, and miRNA systems are operated by substantially overlapping sets of proteins [8], the prokaryotic systems seem to be completely independent from one another.

Clearly, our interpretation of the probable functions of CASS is based, in large part, on the analogy with the eukaryotic siRNA system. There might be danger in heavily relying on analogy as a prediction method because, if the basic premise is false, the entire scheme will fall apart. However, in the case of CASS and eukaryotic siRNA, the analogy stems from two *a priori *independent lines of evidence, namely, the discovery of CRISPR inserts homologous to phage and plasmid genes and the functional similarity between Cas proteins and components of eukaryotic RNAi systems, e.g., dicer. Should there be no *bona fide *functional analogy between CASS and RNAi, there would be no basis for this congruence. By contrast, the homology of CRISPR inserts to phage and plasmid genes and, more generally, the association of *cas *genes with CRISPR have no explanation in the context of our previous hypothesis that CASS is a repair system [23] which forces us to abandon this interpretation of the CASS function.

All the analogies notwithstanding, the predicted psiRNA system shows at least one fundamental difference from the eukaryotic counterpart: the coding segments for the putative psiRNAs are derived from genes of invading agents and incorporated into the host genome to confer heritable immunity to the respective agent. As an acquired immunity mechanism, CASS resembles more the vertebrate immune system than the eukaryotic RNAi pathways but, again, with the crucial difference that the animal immunity is not inheritable. Furthermore, the wide spread of CASS that spans a great variety of prokaryotic lineages contrasts the narrow presence of classical immunity which appears to be a mechanism specific to jawed vertebrates; the recent discovery of a dramatically different immunity system in jawless vertebrates [57] emphasizes the status of the immune system as a lineage-specific evolutionary novelty. More generally, it appears that CASS is one of the most ancient if not, indeed, the primordial biological defense system that probably emerged at an early stage of prokaryotic evolution, considering that diverse viruses, in all likelihood, have accompanied cellular life from its very beginning. Given the ubiquity of CASS in archaea and its less prominent presence in bacteria, one scenario is that CASS emerged in an ancient ancestor of archaea and spread to bacteria horizontally.

Interestingly, as a mechanism of inheritance of acquired traits, CASS seems to come closest to a true Lamarckian mode of evolution among all known systems of heredity. Remarkably, however, this putative system of Lamarckian inheritance appears to be extremely volatile on the evolutionary scale as indicated by the lack of conservation of the psiRNA sequences even between closely related strains. A general implication of this aspect of CASS evolution is that the diversity of mobile replicons (phages, plasmids, transposons etc) in nature might be even more enormous than it is currently estimated [58,59] such that even closely related bacteria occupying similar niches are predominantly invaded by different agents. Additionally or alternatively, it is conceivable that, in many niches, the dominant phages and plasmids rapidly turn over with time, making existing CRISPR cassettes obsolete as defense means and triggering their exchange.

Finally, a practical note. It seems that, once the psiRNA mechanism described here is investigated experimentally, it could be exploited to silence any gene in organisms that encode CASS. The simple design of such experimental gene silencing in prokaryotes will involve transfection with a plasmid containing the desired psiRNA inserted between CRISPR to facilitate homologous recombination.

## Methods

### Genome sequences, databases and sequence analysis

The complete bacterial and archaeal genome sequences were retrieved from National Center for Biotechnology Information (NCBI, NIH, Bethesda) FTP site. The non-redundant database of protein sequences at the National Center for Biotechnology Information (NIH, Bethesda) was iteratively searched using the PSI-BLAST program [31]. The cut-off of E < 0.01 was normally employed for inclusion of sequences in the position-specific weight matrices. Each retrieved sequence was used as the query for additional searches until no new sequences could be detected. For detecting subtle sequence conservation, the PSI-BLAST search results were visually examined and sequences with greater E-values, but containing signature motifs of a given protein family were included into profiles on a case by case basis [30,60]. Multiple alignments of protein sequences were constructed using the MUSCLE program [61] and corrected on the basis of PSI-BLAST results. Protein secondary structure was predicted using the JPRED program [62]. Protein structure comparisons were performed using the DALI [63] and VAST [40] programs, and ribbon diagrams of protein structures were generated made using the program BOBSCRIPT [64].

### Phylogenetic analysis

Distance trees were constructed from multiple protein sequence alignments after excluding poorly aligned positions, by using the least-square method as implemented in the FITCH program of the PHYLIP package [65,66]. Maximum likelihood trees were constructed using the ProtML program of the MOLPHY package, with the JTT-F model of amino acid substitutions, by optimizing the least-square trees with local rearrangements [67,68].

### Identification and analysis of CRISPR repeats

Search for repeats was performed as follows: first, all exactly matching 20-mer anchor substrings were identified in the nucleotide sequence of a bacterial or archaeal genome. Alignments around these anchors were expanded in both directions, to include all adjacent positions with information content above or equal to 1.5 bits [69]. All identified high-similarity fragments were used as queries in nucleotide BLAST [70] search (word size 7; mismatch penalty -1; both gap opening and extension costs -1; E-value threshold 0.001) to detect more diverged versions of the repeats as well as the instances of the repeat in the opposite strand. To determine the repeat boundaries more precisely, sequences of all loci with short (20 nt) flanks added were collected and aligned using the MUSCLE program [61]. Alignments were trimmed from the 5' and 3' termini up to columns with information content exceeding 1.3 bits. Repeat families that shared the spatial arrangement typical of CRISPR (median repeat length of 20–50 nt; median spacer length of 15–60 nt) were identified as CRISPR candidates and further examined for chromosomal proximity to cas genes. The custom PERL scripts used for this analysis are available upon request.

Sequences of eukaryotic miRNAs precursors, CRISPRs, and randomly shuffled CRISPR sequences were computationally folded, and the free energy of the most stable secondary structure was calculated using a a dynamic programming algorithm that employs nearest neighbor parameters to evaluate free energy [71]. Energy minimization was performed by dynamic programming method that finds the secondary structures with the minimum free energy by summing up the contributions from stacking, loop length, and other structural features, using improved thermodynamic parameters [72].

### Similarity of inter-CRISPR spacers to other sequences

Nucleotide sequences of inter-CRISPR spacers were used as a query in MEGABLAST [70] searches (word size 11; e-value threshold 0.01) against GenBank; hits to virus or plasmid sequences and to distantly related prokaryotes were counted separately for each source organism.

## Reviewers' comments

### Reviewer's report 1

Eric Bapteste, Department of Biochemistry and Molecular Biology, Dalhousie University, Halifax, Nova Scotia, Canada

Main comments:

The scientific quality of this paper and its methodology is certain. There has been a lot of good and interesting work done here. As indicated by its title, it thus provides multiple information and one hypothesis about RNA-interference in prokaryotes. Because of this broad scope, the manuscript is quite large. In fact, several of its parts could be read on their own, depending on the reader-specific interest, this is notably the case for the part dealing with the hypothesis of a RNAi prokaryotic immune system. I would thus suggest that a shorter version of the paper, centered around this very interesting hypothesis could be proposed online (with the first part turning into Supp. Mat.), because I feel that this part is going to receive more attention anyway, and it would be unfortunate if some readers did not consider this aspect because they are scared by the overall size of the paper. But this is simply a suggestion, and the authors are more than welcome to disregard my opinion.

**Author response: ***While we fully understand the sentiment and agree that the hypothesis on prokaryotic RNAi is of greater general interest than the detailed presentation of the protein sequence-structure analysis, we strongly feel that the latter provides a badly needed foundation for the hypothesis as well as important information in its own right. Furthermore, we tend to believe that the general spirit of online publication is to present complete results of a study (of course, there are exceptions). The reader can easily navigate between sections, so the length of a paper does not represent a particularly severe problem. Furthermore, we made certain modifications to the protein analysis part in response to similar but more specific comments of Martijn Huynen, in particular, introduced additional subheadings which, hopefully, makes this part of the paper more reader-friendly*.

The seductive hypothesis of a RNAi based immune system is presented as an analogy with the eukaryotic RNAi system. The use of analogy is potentially challenging: on the one hand, it allows a powerful and elegant presentation of many complex genomic results, but on the other hand, it is questionable, since the analogy may impose an a priori model to interpret biological features, and if this model is incorrect, if the analogy does not hold, there is a risk that the genomics data receive a fairly biased interpretation. In this respect, it would be interesting if the authors discuss whether an homologous immune system could have been possible in eukaryotes and in prokaryotes, and why it is not found. Indeed, such an homologous system would be a more natural reference to interpret the data than an analogy.

**Author response: ***We understand the epistemological concerns regarding the role of analogy in this study. However, as indicated by the reviewer himself, the analogy is strong. Moreover, this analogy is manifest at two different levels: i) the presence of inserts homologous to phage and plasmid genes in CRISPR units and ii) presence of predicted activities compatible with a siRNA-like system among cas gene products, in particular, the dicer analog. Had the analogy been false, there would be no reason whatever for this congruence. We briefly comment to that effect in the revised manuscript. The idea of homologous immune systems in prokaryotes and eukaryotes seems a little far-fetched. Nevertheless, this comment prompted us to incorporate a brief comparison of the evolutionary histories of RNAi and classical immune systems*.

This being said, I do not feel that the use of the analogy was a problem here, as it is convincingly presented and argued by the authors. We could eventually question more if all the so-called CASS genes are really involved in the prokaryotic immune systemand deserve their label: some may just be present in the genomic proximity of CRISP, yet having nothing to do with the RNA interference. They might just be mobile «travelling» genes. A further study of the genomic distribution of the homologs of the CASS genes in bacterial genomes may help to clarify which genes are strongly and exclusively CRISP related and which ones can be found also in alternative locations in different genomes. Then, perhaps the «striking diversity of still poorly characterized CASS components» described on page 16 would appear less striking if some CASS categories are simply not relevantly defined, and include unrelated proteins, since maybe, the use of the analogy would had led to too relaxed definitions of CASS. In another situation, by contrast, the use of the analogy could perhaps be too strict. On page 18, the authors wonder how to identify «the slicer counterpart (p-slicer)» in prokaryotes. They explain that this identification is «less straightforward because of the diversity of predicted nucleases within the CASS». But, after all, why should there be only one p-slicer, as in eukaryotes? It is possible that prokaryotes have multiple «p-slicers».

**Author response: ***Yes, we agree, the possibility of multiple slicers exists, and we modified the text to acknowledge this. With regard to the rest of this comment, however, we feel that the current diversity of prokaryotic genomes is already sufficient to make conclusions on the strength of the association of individual genes with CASS, and the genes are classified here accordingly, as true CASS components and loosely associated "satellites". As far as the latter are concerned, inferences on involvement with CASS functions are made only for those genes whose activities appear clearly relevant, like the RT or Argonaut*.

Finally, the strong suspicion about the analogous and intricate functions of COG1518 and COG1343 (cf. page 21) could be similarly toned down. Maybe these genes do play the essential analogous role of CRISPR integrase/recombinase consistently with the analogy, but maybe they fulfill several different tasks. Perhaps the authors would like to comment more on some of these minor points.

**Author response: ***Actually, this prediction is not based on analogy with eukaryotic RNAi systems but rather on the mutualistic association of these genes to CRISPR and the features of the proteins themselves. We dropped the "strong" suspicion but, generally, we strongly believe that this is the best possible prediction. Reference to multitasking might not be particularly productive unless there are good ideas regarding what these multiple functions might be (this is different from the above possibility that there are multiple slicers which is, indeed, compatible with the data)*.

Also, to go back to the CASS gene evolution, the authors mention, page 8, « the extraordinary evolutionary mobility of CASS». It is unclear to me how this statement has been tested, and how the authors have established that CASS genes are more mobile than any average gene randomly picked in the same collection of prokaryotic genomes. For this reason, I am not sure if, as claimed by the authors, on page 14 «the pol-cassette comprises a distinct evolutionary unit that is often transferred horizontally independently of the CASS-core». Does the CASS-core really have an established vertical mode of inheritanceor, as the authors stated before, a «non-uniform» distribution (cf. page 8)? This might be more strongly argued.

**Author response: ***Several distinct issues are addressed here. With regard to the 'extraordinary mobility' of CASS, this is demonstrated by the trees in *Fig. 2*(more trees have been published previously in our own 2002 paper and by Haft et al.), but even more convincingly, by the persistent pattern of presence-absence of CASS in closely related species and even strains of bacteria. We consolidated the argument such that this becomes clear the first time "extraordinary" mobility comes up. It is true that we did not compare the mobility of the CASS components with that of garden-variety prokaryotic genes in a rigorous, quantitative manner. While this is doable, in principle, all methods we are aware of are open to debate, and we feel that the exercise is beyond the scope of this paper. Given the above argument, we believe that, qualitatively, it is clear that CASS is unusual in this respect. With regard to the pol-casette, we believe that the discrepancy between the topologies of the two trees in *Fig. 2*is quite sufficient for the statement on independent HGT. As for the "vertical mode of inheritance" of CASS, there seems to be a semantic issue here. We do not really claim vertical inheritance for CASS but neither is such a pattern necessary to detect horizontal mobility. What is required is a predominant pattern of vertical inheritance among other genes that allows us to use a species tree to detect HGT. Of course, we realize that there are substantial arguments for abandoning "tree thinking"*[73]*altogether but, on balance, we still believe that a species tree conceptualized as a central trend in the evolution of gene ensembles *[74]*is, at least, a useful tool for analysis of genome evolution*.

These few questions show a strength of the present work which interestingly opens perspectives and suggests that some additionnal analyses should now be conducted, because the topic deserves consideration. Maybe the authors would feel like addressing some of the points below in a revised version of the current paper, or in future analyses.

**Author response: ***These are, indeed, very interesting questions, we appreciate them. Some are for future studies but we can provide certain answers now*.

Further study could iclude the following:

-Do other genomic regions harboring concentrations of nucleasescomparable to the ones around CRISP exist elsewhere in the genomes?

**Author response: ***Hardly. As indicated in the paper, the CRISPR neighborhood is the second most prominent (i.e., the one that ranks second in the number of genes) neighborhood in prokaryotic genomes after the ribosomal superoperons, so it is quite outstanding. However, there are other, considerably small constellations of nucleases, such as the classical recBCD operon encoding repair proteins, some restriction-modification systems, and, perhaps, others that are still poorly understood and deserve investigation*.

- If yes, is there more than one prokaryotic immune system definable on this analogous ground? Notably, did bacteria without CRISPR evolve a totally different immune system?

**Author response: ***There is no evidence of that. Furthermore, as repeatedly emphasized in this paper, CASS shows extreme evolutionary volatility, apparently being lost quite easily, in a short time, on evolutionary scale. It is hardly imaginable that these bacteria evolved a distinct immune system in the short time elapsed since the loss of CASS. Of course, purely hypothetically, one could perceive the possibility that another immune system is disseminated horizontally, like CASS, and prokaryotes having both, could differentially lose one of them. However, we are unaware of any support for such a scenario. Another prominent prokaryotic defense mechanism is restriction-modification; it would be interesting to examine the relationship between RM systems and CASS, that could be a subject for a future study*.

-How did the psiRNA pathway arise in thermophiles(cf. page 20)? Does it result from a transfer? Was it ancestral?

**Author response: ***Very interesting, fundamental questions, indeed. In response, we expanded the discussion of these and other aspects of evolution of CASS. The specific preponderance of CASS in thermophiles, noticed already in the 2002 paper, when we thought that this was a thermophile-specific repair system, remains a mystery. Whatever the nature of this association, it seems likely that CASS is ancestral in thermophiles (at least in hyperthermophiles)*.

- Could we imagine that multiples promoters exist, both sense and antisense, which would activate the transcription of CRISPR, generating even more RNAi(cf.p 24)?

**Author response: ***In principle, existence of multiple promoters cannot be ruled out. However, the leader sequence seems to be the only natural candidate for the promoter function. The rest of the CRISPR cassette is homogeneous (repetitive), so it is unclear where an alternative promoter would be located. Further, in the two archaeal systems that have been studied experimentally (Archaeoglobus and Sulfolobus) all transcription of CRISPR loci appear to be unidirectional*.

-Finally, it might be challenging, though interesting to test in vitro on bacterial cultures if, as proposed by the authors, the presence of CRISP and CASS, has really an impact on the fitness of prokaryotes in presence of viruses.

**Author response: ***We certainly hope that the computational analyses and predictions described in this paper stimulate a lot of experimentation aimed at elucidation of the biological functions of CASS and roles of its individual components*.

*We greatly appreciate these insightful and stimulating comments*.

Minor comments/questions:

On page 7: «functionally analogous» is redundant.

**Author response: ***We see the point but do not really agree. The word "functionally" seems to add clarity*.

On page 8: the sentence «the distribution of COG1518 and, by implication, CASS among prokaryotic lineages...» is too «bold» for me: even if the conclusion is correct, I am not sure one can generalize as suggested here from the case of one protein only.

**Author response: ***Indeed, we can. Rephrased to clarify and emphasize this*.

On page 15: «several other CASS gene families remain mysterious» is a mysterious sentence. I am not sure what this does really mean.

**Author response: ***That there is no clue as to the possible functions of these proteins; modified to clarify*.

On page 21: I miss the idea of the sentence starting by «In addition, and probably, more relevantly etc.» to «retroviral genomes». Could you rephrase it to explicit it a little bit more?

**Author response:***Rephrased – hopefully, to clarify*.

On page 23: what is the criterion retained for homology between the plasmid genes, fragments of phages and the CRISPR sequences?

**Author response: ***The following quote from the Methods addresses this issue:*

*"Nucleotide sequences of inter-CRISPR spacers were used as a query in MEGABLAST *[70]*searches (word size 11; e-value threshold 0.01) against GenBank; hits to virus or plasmid sequences and to distantly related prokaryotes were counted separately for each source organism."*

On page 48: To me, the multiple positive correlations evoke multiple causalities and the possibility of some hidden correlations. Would you say that all the relevant combinations have been considered here?

*No, we won't claim that. More complex multiple regression analysis would be required to separate correlations that reflect true causality; for the purposes of this paper, we felt it was sufficient to note the strongest correlations*.

### Reviewer's report 2

Patrick Forterre, Biologie Moléculaire du Gène chez les Extrêmophiles (BMGE) Institut de Génétique et Microbiologie (IGM), Université Paris-Sud, Centre d'Orsay, 91405 Orsay Cedex, France, and Biologie Moléculaire du Gène chez les Extrêmophiles (BMGE), Département de Microbiologie Fondamentale et Médicale, Institut Pasteur, Paris, France

In this very important paper, Makarova and coworkers propose a detailed mechanism for a putative procaryotic antiviral immunity system mediated by CRISPS sequences and their associated Cas proteins (the CAS system, CASS *sensu *the authors). Their model is based on the hypothesis that these elements represent a prokaryotic-specific antiviral mechanism analogous to the eukaryotic RNAi system. In procaryotes (Bacteria and Archaea) there is no homologs of the proteins involved in the eucaryotic RNAi system. Untill recently, it was therefore widely believed that restriction-modification mechanisms were the only defense available to procaryotes to fight viral infections. However, it has been proposed last year by several groups that procaryotic CASS could also play a significant role in fighting viral aggression in archaea and bacteria (Mojica et al. 2005, Pourcel et al., 2005, Bolotin et al., 2005). CRISPR sequences, which are transcribed but non-coding, are formed by the tandem repetition of units containing both a conserved element (similar all along a given CRISPR) and a variable element, the spacer, different from one unit to the other. The spacer sequences have strikingly no homologous sequence in databases, except for viral or plasmid sequences. Both Mojica et al. (2005) and Bolotin et al., (2005) have suggested that transcription of CRISPR sequences produce anti-sense RNA that can inhibit transcription of incoming viral (plasmid) sequences and Mojica et al. (2005) mentioned the analogy of such system with eukaryotic RNAi. However, these authors did not elaborate on the specific mechanism involved and how the cas proteins could be involved in the processing of viral RNA.

In this work Makarova and co-workers have first performed an updated analysis of cas proteins using genomic context analysis and sensitive methods (iteration approaches) to detect low level of similarity and to classify cas proteins in families and superfamilies. They were able to identify several new putative cas proteins and to define 25 superfamilies of cas proteins and 7 different types of CASS organization (named CASS1 to 7). They have also analyzed all available CRISPR repeated sequences and their putative secondary structures. More importantly, they try to predict the biological function of the cas proteins and their mechanism of action in the framework of the RNAi hypothesis. Previously, it has been suggested that cas proteins were involved in the formation and spreading of the CRISPR. For instance, Bolotin et al. Have predicted that cas proteins are acting at the DNA level by promoting cleavage, recombination and ligation. Makarova and al are the first to suggest that several cas proteins should instead interact at the RNA level, by promoting RNA degradation and RNA-RNA hybridization. They specifically suggest the existence of procaryotic homologs of eucaryotic dicer (helicase-nuclease) and splicer (nuclease). They also propose that a previously suspected DNA polymerase could be an RNA dependent RNA polymerase used to stabilize RNA/RNA hybrid by extending iRNA hybridized to their viral mRNA target. They also suggest the involvement of a reverse transcriptase in the formation of the linker sequences from viral (plasmidic) mRNA. In my opinion, all these proposals are reasonnable and very convaincing. Another prediction is that RAMP proteins recognize linker sequences of different sizes. This is supported by a correlation between the number of linker sequences and the number of RAMPs encoding genes (Fig. 6). In that case, it's not clear to me why this could not be due to the binding of RAMPs to the repeated units, since these units exhibit conserved sequences and their number (identical to the number of linkers) should be also correlated with the number of RAMPs.

**Author response: ***That RAMPs discriminate, one way or another, between CRISPR inserts, is strongly suggested by the extreme sequence divergence of RAMPs which is hardly compatible with recognition of identical repeats. To be explicit about it, we added a clarification at the end of this section*.

The search for specific secondary structure associated to the repeated units did not give convincing results and suggest for me that the dyad symmetry observed in many repeat units could be due to the binding of proteins with repeated structure (possibly the duplicated ferredoxin-like fold present in RAMPs) and not the formation of secondary structures in the transcribed repeats.

**Author response: ***It is hard to see how one excludes the other: it stands to reason that CRISPR do form distinct secondary structures which bind to symmetrical proteins*.

The model proposed (including possible variation) thus implies many predictions that could be experimentally tested. Surprisingly, to my knowledge, only one cas protein has been studied at the bench up to now (ref 70 in the manuscript). This protein turns out to have DNAse activity *in vitro*, but I suspect that the authors have not tested a possible RNAse activity. This is surprising because the importance of these proteins was already highlighted in 2002 by two *in silico *papers that in one case suggested their participation to a "mysterious DNA repair system and in the other described their association with CRISPR sequences. The present paper, with much more specfic predictions, should hopefully strongly stimulate biochemists and molecular biologists to jump onto this really exciting story. As noticed by the authors in their conclusion, if their hypothesis turned out to be correct, this prokaryotic RNAi system could be exploited to silence any gene in organisms that encode CASS. Furthermore, the experimental study of this system should help us to get new critical insights on the dynamic relationships between viruses and archaeal/bacterial populations in nature.

Finally, I would like to know if the authors have some idea about the origin of this CAS system. Why is it present in all archaeal genomes sequenced so far? Is it possible that this system originated in Archaea and was later on introduced in bacteria by LGT?

**Author response: ***Given the horizontal mobility of CASS, we can only speculate on the point of its origin. We expand such speculation in the revised conclusion including the possibility of archaeal origin*.

- In some case, the authors should be more cautious in their statement. For instance, when they talk about the pol-cassette, it might led some reader to believe that the polymerase actvity of the COG1353 protein has been experimentally validated, which is not the case.

**Author response:***We added a few more "predicted". However, we did not want to abandon the term 'pol-cassette' as it is descriptive and succinct*.

### Reviewer's report 3

Martijn Huynen, Nijmegen Center for Molecular Life Sciences University Medical Center St. Radboud p/a Center for Molecular and Biomolecular Informatics, Nijmegen, Netherlands

This paper provides a highly interesting and well documented hypothesis about a cluster of genes that E. Koonin and co-workers have discovered some time ago. By combining biological knowledge with bioinformatics methods and creative thinking the authors propose that Archaea and to a bit lesser extent Bacteria posses an RNA-interference-based immune system involving CRISPR and cas genes, that is analogous the eukaryotic RNA interference systems. Although aspects of this hypothesis have been published before, specifically with respect to CRISPR, this paper is, as far as I can tell, the first that makes the analogy between the cas genes and the RNA interference system. The idea that prokaryotic genomes would internalize pieces of foreign DNA in order to be able to defend themselves against it, thus having an immune system with a memory, would be an interesting example of Lamarckian evolution.

I do have some questions and editorial comments that I think should be addressed.

1) Do the authors have any idea why this system has the phylogenetic distribution that it does, being present in such a small genome as the nanoarchaeon, but not in e.g. the majority of Firmicutes

**Author response: ***No mechanistic idea, unfortunate as this might be. We added some additional discussion of the ultimate origin of CASS (see the response to Patrick Forterre)*.

2) concerning the feasibilty of the system proposed by the authors: Is there anything known about how many fiendly DNAs a prokaryote encounters in daily life, and how does that compare to the number of different elements in a CRISPR ?

**Author response: ***Not enough for this particular comparison. However, it is well known that phages are extremely abundant, much more so than bacteria or archaea, and in the revised manuscript, we refer to this more specifically, with the corresponding references*.

3) Regarding the Lamarckian scheme: That the unique element of the CRISPR correspond to highly conserved, essential elements of phage genes suggests that selection on genetic variation also plays role here. So the scheme would be partly Lamarckian.

**Author response: ***Probably, so. The way we state it in the text "CASS seems to come closest to a true Lamarckian mode of evolution among all known systems of heredity" is compatible with this view*.

4) I am not so convinced by the argument on page 3 that the results imply that even among closely related prokaryotes the most commonly encountered phages are different. First of all, it is more a corollary of the hypothesis, but second, it could also reflect the high turnover of phages over time, rather than niche.

**Author response: ***This is a very good idea, we now mention this possibility both in the Abstract and in the Discussion*.

5) I am puzzled on the involvement of more or less randomly selected pieces of DNA from foreign DNA/RNA in exactly the same location in the secondary structure of the psiRNA (the top of the hairpin). Does this pattern occur more often?

**Author response: ***The situation when the insert forms a stem of varying stability with parts of the repeats is common but not universal. The positions of the inserts are not exactly the same although they are, indeed, very similar, and the stems in which the inserts are involved are imperfect. Of course, the exciting possibility exists that the CRISPR inserts are specifically selected for their ability to base-pair with the repeats, however, we do not have enough data to make that claim*.

## Authors' contributions

KSM performed the protein sequence analyses, NVG performed protein structure comparisons and modeling, SAS performed RNA secondary structure predictions, YIW performed nucleotide sequence analysis, EVK developed the biological interpretations of the results and wrote the manuscript which was edited and approved by all authors.

## Supplementary Material

Additional file 1 GI number for all families of Cas proteins. Click here for file

Additional file 2 multiple protein sequence alignment for COG1343. Click here for file

Additional file 3 multiple protein sequence alignment for COG1857. Click here for file
